# Seroprevalence and risk factors for lumpy skin disease virus seropositivity in cattle in Uganda

**DOI:** 10.1186/s12917-019-1983-9

**Published:** 2019-07-08

**Authors:** Sylvester Ochwo, Kimberly VanderWaal, Anna Munsey, Joseph Nkamwesiga, Christian Ndekezi, Elda Auma, Frank N. Mwiine

**Affiliations:** 1 College of Veterinary Medicine, Animal resources and Biosecurity, Makerere University, P.O.BOX 7062, Kampala, Uganda; 2 College of Veterinary Medicine, University of Minnesota, 1365 Gortner Avenue, St. Paul, MN MN 55108 USA; 3 College of Natural Sciences, Makerere University, P.O.BOX 7062, Kampala, Uganda

**Keywords:** Lumpy skin disease, Seroprevalence, Risk factors, Uganda

## Abstract

**Background:**

Lumpy skin disease (LSD) is a transboundary cattle disease caused by a *Capripoxvirus* of the family *Poxviridae*. In Uganda, documented information on the epidemiology of the disease is rare and there is no nationwide control plan, yet LSD is endemic. This study set out to investigate the seroprevalence of lumpy skin disease and determine the risk factors for LSD seropositivity, by carrying out a cross-sectional study in 21 districts of Uganda.

**Results:**

A total of 2,263 sera samples were collected from 65 cattle herds and an indirect ELISA was used to screen for lumpy skin disease virus (LSDV) antibodies. We used univariable and multivariable mixed effect logistic regression models to identify risk factors for LSD seropositivity. The overall animal and herd-level seroprevalences were 8.7% (95% CI: 7.0–9.3) and 72.3% (95% CI: 70.0–80.3), respectively. Animal-level seroprevalence in Central region (OR = 2.13, *p* = 0.05, 95% CI: 1.10–4.64) was significantly different from the Northern region (Reference) and Western region (OR = 0.84, *p* = 0.66, 95% CI: 0.39–1.81). Management type, sex, age, mean annual precipitation > 1000 mm, and drinking from communal water sources were statistically significant risk factors for occurrence of anti-LSDV antibodies in cattle. Breed, region, herd size, contact with buffalo and other wildlife and introduction of new cattle did not have a statistically significant association with being positive for LSDV.

**Conclusion:**

We report a high herd-level LSDV seroprevalence in Uganda with a moderate animal-level seroprevalence. Cattle with the highest risk of LSD infection in Uganda are those in fenced farms, females > 25 months old, in an area with a mean annual rainfall > 1000 mm, and drinking from a communal water source.

**Electronic supplementary material:**

The online version of this article (10.1186/s12917-019-1983-9) contains supplementary material, which is available to authorized users.

## Background

Lumpy skin disease (LSD) is a transboundary disease of cattle. The etiological agent of LSD is a double stranded DNA *Capripoxvirus* which belongs to the family *Poxviridae* [1, 2]. This disease is defined by mild to severe symptoms which include fever and development of large nodular skin and internal organ lesions [3–6]. These symptoms lead to death in 1–5% of the cases [7]. Lumpy skin disease virus (LSDV) is closely related to two other viruses in the genus *Capripoxvirus*, sheeppox and goatpox viruses [5]. Transmission of LSDV is primarily by mechanical means, by several probable arthropod vectors such as biting flies, mosquitoes (*Aedes aegypti*) [8] and three tick species of the family Ixodidae, namely *Rhipicephalus appendiculatus, Rhipicephalus decoloratus and Amblyomma hebraeum*) [9]. These vectors have been shown to transmit LSDV under experimental conditions, however their capacity to transmit disease under natural field conditions is still unknown. *Stomoxys calcitrans* (Stable fly) has been reported as the most probable vector for LSDV due to its abundance and being associated with outbreaks [10, 11]. Veterinary equipment, specifically needles [5, 12] have also been reported to transmit the virus. The possible vectors of LSDV (mosquitoes, flies, ticks) are all present in Uganda and are highly likely to be responsible for disease spread, although the contribution of different vectors in LSDV transmission has not yet been studied in the country.

Morbidity of LSD varies greatly and ranges from 3 to 85% in different epizootic situations. In endemic areas, morbidity is estimated at 10% [5]. Mortality due to LSD varies between 1 and 3%, but up to 40% have been reported in severe outbreak situations [7]. These broad ranges for morbidity and mortality are likely to be due to variation in cattle breed, health status, viral isolates and insect vectors involved in the transmission [4, 13]. For instance in Africa, imported breeds from Europe or Australia have shown high susceptibility to LSD [13–15].

Control and prevention of LSD is undertaken through vaccination, quarantines, livestock movement controls, vector control, slaughter of infected and exposed animals and cleaning and disinfection of the premises [5]. Vaccination is reported to be the most effective method for controlling LSD in both disease endemic and non-endemic areas [16]. At present, only live vaccines are commercially available against LSDV, with different vaccines licensed for use in different countries although the most common LSDV strain used in attenuated vaccines is the Neethling strain. This strain was reported to be highly effective in controlling epidemics in the Balkans [17]. In countries where goat pox is present, attenuated goat pox virus strain can be used, like the Gorgan goat pox strain. In Uganda, two Neethling virus based vaccines, BOVIVAX LSD-N and LUMPYVAX™ are licensed for use in the country [18]. Vaccination against LSD is primarily done by commercial farmers who can afford the costs of vaccination while small holder farmers largely do not vaccinate against LSD.

Lumpy skin disease was first reported in northern Rhodesia (Zambia) in 1929 [19] and the disease remained endemic to sub-Saharan Africa until 1990, when it extended into North Africa and then into the Middle East [20, 21]. More recently, LSD has spread into parts of southeast Europe, with outbreaks reported in Turkey and Russia amongst other countries [22–26]. This disease therefore has the potential for global emergence [27]. In Uganda, there is no published literature about when LSD was first identified. The disease is thought to have spread from southern Africa into Uganda between 1955 and 1960 [28]. The disease (LSD) is currently present in all geographical regions of the country, with several outbreaks reported annually. Suspected outbreaks are reported based on clinical signs and are confirmed by the National Animal Disease Diagnostics and Epidemiology Centre (NADDEC) Laboratories, using polymerase chain reaction (PCR)-based methods [29]. Following LSDV confirmation, control is mainly by isolation of infected animals from the herd and treatment of secondary infections with injectable antibiotics and non-steroidal anti-inflammatories (NSAIDs). Occasionally, vaccination of animals is carried out in the affected herds and in neighboring herds to prevent further spread of disease. These vaccinations are usually self-funded by the farmers and are therefore based on availability of vaccines, interest in pursuing vaccination and on financial ability of farmers. This therefore limits the number of animals that get vaccinated. Vaccination against LSD is mainly done by commercial farmers with large herds while small holder farmers, which constitute the vast majority of livestock holders, do not vaccinate against LSD.

A number of methods have been used for serological investigation of LSDV. These tests include a skin hypersensitivity test [3], virus neutralization test (VNT), immunoperoxidase monolayer assay (IPMA) [30] or indirect fluorescent antibody test (IFAT) [31] and ELISA. The antibody ELISAs developed in the past have faced challenges like difficulty in obtaining sufficient quantities of heat inactivated antigen and instability of recombinant antigens [32, 33]. This had therefore led to the inability to do mass screening of LSD using a reliable method. This study utilizes the first commercially available ELISA for detection of LSD antibodies in naturally infected and in vaccinated animals. This ELISA, ID Screen® Capripox double antigen multi-species ELISA, is able to detect antibodies against Capripoxviruses including lumpy skin disease virus (LSDV), sheeppox virus and goatpox virus in serum and plasma. It has demonstrated very high specificity (> 99.7%) and does not cross-react with parapox viruses. It also has equivalent sensitivity to immunoperoxidase monolayer assay (IPMA) [34], when compared to VNT and IFAT respectively. This ELISA is easy to perform and allows for high throughput screening without requiring high-level containment facilities [35].

In Uganda livestock farming contributes 1.7–3.2% of GDP [36], however growth in this sector has been slow because of livestock diseases. LSD is one of the many important cattle diseases in Uganda, but unlike other cattle diseases in the country, little is known about the epidemiology of the disease and as a result there is presently no control strategy. LSD is usually reported by farmers and district authorities and is enzootic in all regions of the country. In order to understand more about the epidemiology of LSD in Uganda, a recent retrospective study assessed the spatiotemporal distribution of reported LSD outbreaks [37]. This study therefore seeks to provide more epidemiological information about LSD in Uganda by estimating the herd- and animal-level seroprevalence, and risk factors for seropositivity, in selected herds with no history of vaccination against LSD, within all four major geographical regions of Uganda. This will help in adding to the scarce epidemiological knowledge of LSD in Uganda and potentially contribute to better control measures for the disease.

## Results

### Demographic characteristics of the study population

Blood samples collected from 2,263 cattle in 21 districts of Uganda between July 2016 and August 2017 were screened for presence of antibodies against LSDV. The average number of cattle sampled per region was 565.8 while the mean number sampled per herd was 28.6, distributed over 65 herds. Of the cattle sampled, 1,840 were female while 423 were male. Most of the cattle sampled were of the zebu breed (1,094), followed by Ankole breed (834), Friesian (146), Boran (129) and Friesian-Ankole crossbreeds (60).

### Seroprevalence of LSD antibodies at animal level in Uganda

The overall animal-level seroprevalence of lumpy skin disease in Uganda was found to be 8.7% (95% CI: 7.1–9.4). The highest regional seroprevalence was 12.8% (95% CI: 9.3–15.0) in the Central region, followed by 10.3% (95% CI: 7.3–12.4) in the Eastern region, 6.1% (95% CI: 4.0–8.1) in the Northern region, and 6.2% (95% CI: 4.2–8.0) in Western region respectively. The differences in animal level prevalence amongst these four regions were found to be statistically significant, with there being a statistically significant difference in seroprevalence between the Central region (OR = 2.13, *p* = 0.05, 95% CI: 1.10–4.64) as compared to the Northern region.

### Herd-level prevalence

Among the 65 herds investigated in this study, 47 (72.3%) of the herds had at least one seropositive animal for LSDV. Herd-level seroprevalence at the regional level was 100% for the herds sampled in Central and Eastern Uganda, while the north and west had herd-level prevalences of 69.3 and 59.4%, respectively. Within-herd seroprevalence varied between 0 and 35.6%, with an average within herd seroprevalence of 7.7%. Prevalence per sampled herd and at regional level are summarised in Table 1 and Additional file 3.

**Table 1:** Animal- and herd-level seroprevalence of lumpy skin disease in the four major geographical regions of Uganda

Animal-level seroprevalence					Herd-level prevalence			
	Cattle tested	Positive cattle	True Prevalence (%)	95% CI	Herds tested	Positive herds	True Prevalence (%)	95% CI
Region								
Northern	552	32	6.1	4.0–8.1	19	12	69.3	41.0–81.0
Central	527	63	12.8	9.3–15.0	14	14	100	75.0–100.0
Eastern	542	52	10.3	7.3–12.4	8	8	100	63.0–100.0
Western	642	38	6.2	4.2–8.0	24	13	59.4	35.0–72.0

### Risk factors for LSDV seropositivity

Univariable mixed effect logistic regression was performed on the following possible risk factors for LSD seropositivity: Age, management type, sex, breed, mean annual rainfall, region, contact with buffalo, communal water source, new cattle introduced, contact with other wildlife and herd size. Out of the eleven possible risk factors analysed, management type, age, sex, mean annual rainfall, contact with buffalo and communal water sources, and with *p* < 0.2 were selected for multivariable analysis. The best fit model included management type, sex, age, mean annual rainfall, contact with buffalo, and communal water sources as significant factors associated with LSDV seropositivity. As compared to pastoral and communally grazed herds, fenced farms were significantly associated with seropositivity (OR = 5.26; 95%CI: 2.64–10.48, *p* < 0.01). In addition, higher mean annual rainfall (1001-1200 mm-OR = 5.60; 95%CI: 2.35–13.34, p < 0.01; 1201-1400 mm-OR = 4.58; 95%CI: 2.23–9.40, *P* < 0.01), female cattle (OR = 1.72; 95%CI: 1.02–2.92, *p* = 0.04), age > 25 months (OR = 1.96; 95%CI: 1.15–3.34), and drinking from communal water sources (OR = 3.31; 95%CI: 1.42–7.71, *p* = 0.01) were significant factors associated with LSDV seropositivity (Table 2). Daily reported contact with buffalo (OR = 1.78; 95%CI: 0.50–6.31, *p* = 0.37) was found not to be a significant factor for LSDV seropositivity, whereas weekly or monthly contact with buffalo had a protective effect, as shown in Table 2.

**Table 2:** Best-fit multivariable model for animal-level risk factors associated with LSDV sero-positivity using mixed effect logistic regression modelling with herd as random effect

Risk factors	Odds Ratio	95% CI	*P*-Value
Management type			
Communal/pastoral	Ref		
Fenced farm	5.26	2.64–10.48	< 0.01
Zero grazing	0.28	0.06–1.44	0.13
Mean annual rainfall (mm)			
800–1000	Ref		
1001–1200	5.60	2.35–13.34	0.00
1201–1400	4.58	2.23–9.40	0.00
Sex			
Male	Ref		
Female	1.72	1.02–2.92	0.04
Age in months			
0–12	Ref		
13–24	1.24	0.63–2.44	0.54
> 25	1.96	1.15–3.34	0.01
Contact with Buffalo			
Never	Ref		
Daily	1.78	0.50–6.31	0.37
Weekly/monthly	0.49	0.29–0.85	0.01
Communal water source			
No	Ref		
Yes	3.31	1.42–7.71	0.01

## Discussion

In this study we investigated the seroprevalence and risk factors for seropositivity against lumpy skin disease virus in the four major geographical regions of Uganda using the first commercially available antibody ELISA test for LSDV. This is the first study reporting seroprevalence of LSDV in cattle in Uganda. We found an overall animal-level seroprevalence of 8.7% and an overall herd-level seroprevalence of 72.3%.

The overall animal-level sero-prevalence of 8.7% in Uganda is comparable to LSDV prevalences reported by previous studies in the East African region. In Ethiopia, two studies have reported a lower prevalence; one study by Abera et al reported a sero-prevalence of 6.43% [15] while the other study by Hailu et al reported prevalence of clinical LSD at 7.4% [38] in north-eastern Ethiopia. Higher animal-level prevalences have also been reported by Gari et al [39], who estimated a sero-prevalence of 23–31% in different agroecological zones in Ethiopia, and Molla et al [40] who reported a sero-prevalence of 26.5%. Comparison of herd-level sero-prevalence, however, showed a higher prevalence in Uganda (72.3%) than herd level sero-prevalence reported in Ethiopia. Previous studies have reported varying herd-level prevalences in lowland (50%), midland (26%) and highland agro-climate zones (64%) in Ethiopia [39]. Herd-level prevalence was reported as 52.6% in central and northwestern Ethiopia [40] and 5.95% in western Ethiopia [15]. There have been no recent studies on sero-prevalence of LSDV in Uganda’s neighboring countries of Kenya, South Sudan, Congo DRC, Tanzania and Rwanda. This may be mainly be due the fact that the previously available tests for serodiagnosis of LSDV required virus isolation and high levels of biocontainment, which are mostly lacking in these countries. Serum neutralization tests (SNT), immune peroxidase monolayer assays, and indirect fluorescent antibody tests are accurate but have limitations for screening large numbers of serum samples. In addition, many ELISAs developed in the past have not been made commercially available [35].

When we compared animal-level sero-prevalence amongst the four regions of Uganda, the highest sero-prevalence was reported in the Central region (12.8%), followed by 10.3% in Eastern region, 6.2% in Western region, and 6.1% in the Northern region. These differences in animal-level sero-prevalence amongst these four regions were found to be statistically significant, with significantly higher sero-prevalences in the Central region as compared to the Northern region. There was no significant difference between sero-prevalence in the Central and East. These findings suggest that cattle in Central and Eastern Uganda are exposed to LSDV at higher rates than in the West and Northern regions. This could be due to environmental conditions in these two regions being favorable for reproduction of biting arthropods which act as vectors for LSDV and due to differences in husbandry practices. These regional differences in sero-prevalence of LSDV are in agreement with our previous findings which, using retrospective reporting data, demonstrated the highest numbers of clinical cases of LSD in Uganda to be in the Central and Eastern regions [37]. Other than the risk factors investigated in this study, there could be a number of bioclimatic and epidemiological variables responsible for these high prevalences that warrant further investigation. For example, increased unregulated acaricide use and resistance in Uganda may also be playing a role in maintaining high LSD prevalence, since ticks are known to transmit LSD [41].

Across Uganda, 72.3% of herds reported at least one seropositive animal for LSDV. However, all herds tested in Central and Eastern Uganda had at least one positive animal per herd, thus giving a herd-level prevalence of 100%, while the North and West had lower herd-level prevalences of 69.3 and 59.4%, respectively. It is reported that the duration of antibodies in infected cattle is short-lived and declines within 6 months following infection [42, 43]. This high herd-level prevalence, particularly in Central and Eastern Uganda, suggests that the LSDV incidence is frequent and that the virus may be maintained locally within cattle populations.

We additionally studied risk factors for seropositivity for LSDV, and found management type, sex, age, mean annual rainfall as significant factors associated with LSDV in Uganda. Cattle kept in fenced farms (OR = 5.26: 95%CI: 2.64–10.48) showed higher risk for LSD infection than those grazed communally or in a pastoral husbandry system. In fenced farms, cattle are kept in a confined grazing area, potentially allowing for biting flies to easily transmit disease. Higher intensity of range use within fenced farms may also increase transmission of environmentally-transmitted pathogens [44]. Alternatively, fenced farms may be a management system that is common in wetter environments, and thus the significance of fenced farms may be an artifact underlying environmental conditions that are conducive to both higher densities of arthropod vectors as well as fenced farming practices. We also found an association between being female and seropositivity; female animals were almost twice more likely to be seropositive when compared to males. In this study, adult cattle > 25 months were found to have higher odds of seropositivity (OR = 1.96) as compared to young cattle (0–12 months). The reason for higher seropositivity in older animals may be related to duration of exposure, which increases their chance of infection. Similarly, female animals are usually kept longer by farmers while males are sold off at a younger age, thus the effect of sex may be an artifact of duration of exposure. These findings agree with Molla et al [40] (OR = 2.44) and Abera et al [15] (OR = 3.41), both of which recorded higher odds of LSDV in adults compared to calves. This may also be due to low frequency of exposure, because calves are kept at home in small enclosed spaces away from biting insects. In a mathematical modeling study evaluating different routes of LSDV transmission, Magori-Cohen et al [45] reported that suckling calves showed the lowest infection rate, possibly due to their location away from infected adult cattle and biting flies.

Mean annual rainfall ranges of 1001-1200 mm (OR = 5.60: 95%CI: 2.35–13.34) and 1201-1400 mm (OR = 4.58: 95%CI: 2.23–9.40) were found to be associated with LSDV seropositivity as compared to mean annual rainfall less than 1000 mm. High rainfall is associated with increased arthropod density, therefore increasing populations of insect vectors responsible for mechanical transmission of LSDV. Here, we additionally report drinking from communal water sources as a risk factor for LSDV seropositivity. Cattle drinking from a communal water source were three times more likely to be seropositive than those drinking from a non-communal water source. This is in agreement with other studies reporting that animals having frequent contact with other animals at communal grazing and watering points are more at risk to acquire LSDV [38, 46]. We found weekly/monthly contact with buffalo to be protective against seropositivity, while daily contact with buffalo did not have a significant association perhaps due to a small numbers of herds within this latter category. These findings are likely reflective of some unmeasured variable or factor that was not fully captured in this study, such as finer scale environmental gradients in which contact with wildlife served as a proxy for drier conditions .

In this study we compared local Ankole, Boran and Zebu cattle breeds with Friesian and found no association between cattle breed and LSDV seropositivity. This is unlike other studies, by Davies FG [13] and Abera et al [15] who reported Channel Island breeds and Zebu-Friesian cross breeds respectively, as having a higher risk for LSD as compared to local breeds. We also found no association between herd size and introduction (re-stocking) of new cattle to the herds and increased LSDV seropositivity.

## Conclusion

This is the first study on sero-prevalence of LSDV in Uganda and it provides baseline information on the occurrence of LSDV in Uganda. This study reported a high herd-level sero-prevalence of LSDV in Uganda, demonstrating that LSDV is an important disease in Uganda. The overall animal-level sero-prevalence is moderate. This study also confirms that LSDV is prevalent in all regions of the country, with higher rates of exposure in the Central and Eastern regions. Cattle with the highest risk of LSDV sero-positivity were found in an area with a mean annual rainfall > 1001 mm, those in fenced farms, females, cattle older than 25 months, and drinking from a communal water source. Appropriate control measures should be developed with our findings in mind.

## Methods

### Description of study area

**Fig. 1:**
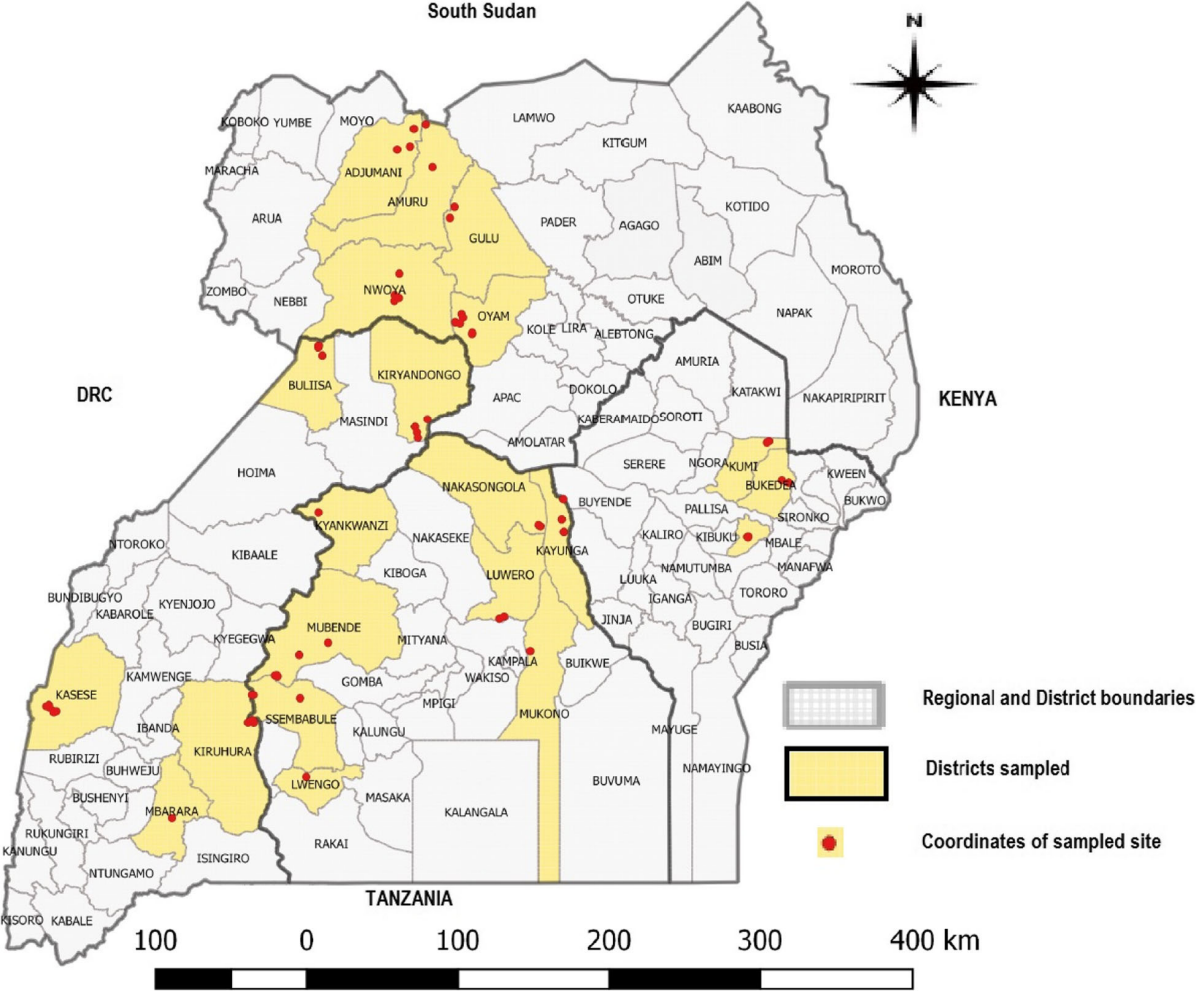
Map of Uganda showing the study districts in yellow and gps coordinates (red dots) of sites where individual cattle herds were sampled (Source of map: This study)

Uganda is an East African country found between latitudes 4°N and 2°S, and longitudes 29° and 35°E, with an area of about 241,038 km^2^. Uganda averages about 3,609 ft. (1,100 m) above sea level, and a large portion of its southern border is lakeshore. The country is mostly plateau with some undulating hills and low mountains. Uganda is divided into four administrative regions: North, East, Central and West, comprising 121 districts. The districts are further divided into counties, sub-counties, parishes and villages. The cattle population in Uganda is estimated to be about 11.4 million heads of which 34.2% are in the north, 21.8% in the East, 21.7% in Central and 22.3% in the west [47]. Within these regions the highest number of cattle are found in districts along the Uganda cattle corridor, which is an area stretching diagonally across the country from the southwest to the northeast. This cross sectional study was carried out in 21 districts of Uganda spread-out in all four regions in Uganda, in herds with no history of LSD vaccination (Fig. 1). These selected areas are comprised of two major livestock farming systems: mixed rainfed crop-livestock systems and livestock-only farming systems. In the mixed rainfed crop-livestock system, crops and livestock farming are practiced together, whereas in the livestock-only, rangeland-based livestock production system, livestock farming is the major type of farming with nearly no crop farming practiced. The livestock-only rangelands are part of the Uganda cattle corridor.

### Study design, sample size determination, and data collection

The first aim was to estimate differences in sero-prevalence across the four regions of Uganda, as the four regions have varying mean annual rainfall and livestock production systems [48]. Target sample size per region was 520, which was based on the sample size needed to detect differences between two proportions with 0.8 power and 0.95 confidence level. We assumed that sero-prevalences in Uganda would fall between estimates from elsewhere in Eastern Africa: 8% [15, 31] and 16% [39]. The sampling frame included the 211 herds that were sampled as part of a nationwide sero-survey for foot-and-mouth disease virus from 2016 to 2017 (Additional file 1). During this sero-survey, information on vaccination against common cattle disease was collected and herds with no history of LSD vaccination were selected for this study. As suggested by Dohoo et al. (2009) for study designs with multistage sampling, herds within each region were selected such that the probability that each herd was selected was proportional to their herd size [49]. Only herds with at least 20 animals were included. Based on these criteria, 2,263 samples from 65 different herds were screened for LSDV. Livestock in this study were indigenous short horned zebu, Ankole, Boran, Friesian and crossbred cattle of all age groups ranging between 2 months to 6 years old. In the course of sample collection, cattle owners were consulted to help estimate the age of each sampled animal. All the sampled cattle were grouped into three age based categories; cattle between 0 and 12 months were considered calves, cattle of 13–24 months were considered young, and cattle older than 24 months were considered adults. At each sampling location, a questionnaire was administered to the animal owner to collect information about possible risk factors at the animal and herd-level. Assistance in administering the questionnaire was provided by area (local) veterinary personnel in the appropriate language/dialect. The GPS of the herd was recorded.

### Sample collection and handling

Full disposable 8.5 ml sterile Vacutainer SST tubes of whole blood samples were collected from the jugular vein of each animal. The tubes were then kept protected from direct sun light at room temperature until the blood clotted and sera were separated by centrifugation at 3000 rpm for 10 min. The separated sera were transferred to sterile 2 ml cryovials, labeled with animal number, age, sex and district name. The samples were then transported to Makerere University, College Of Veterinary Medicine Animal Resources and Biosecurity (COVAB) molecular biology laboratory for serological examination using an indirect ELISA test.

### ELISA test for LSD antibodies

Antibodies against LSDV were detected using a Double Antigen ELISA (ID Screen®) for the detection of antibodies against Capripoxviruses. Briefly, to perform the ELISA test, 50 μL of each test serum sample was diluted in 50 μL of Dilution buffer 19 and added to an ELISA plate coated with Capripox virus purified antigen. Positive and negative control sera were also similarly diluted and added to the same ELISA plate. The ELISA plate was incubated for 90 min at 21 °C, wells were emptied, then washed 5 times with wash solution before adding 100 μL conjugate to each well, followed by incubation for 30 min. Wells were then emptied and washed 5 times, then 100 μL of substrate solution was added to each well and the plate covered and incubated for 15 min. 100 μL stop solution was then added and the optical density (OD) read at 450 nm using a microplate reader (Biochrom Asys UVM 340, UK). For each sample, the percentage OD of sample/ OD of positive control (S/P percentage) was calculated using the formula:

$$ S/P\%=\frac{OD_{sample}-{OD}_{Nc}\ }{OD_{PC}-{OD}_{NC}}\times 100 $$

Where OD sample is the optical density of sample, OD_PC_ is optical density of positive control, and OD_NC_ is optical the optical density of negative control. Samples presenting an S/P% less than 30% were considered negative while those with S/P% greater than or equal to 30% were considered positive.

### Data management and statistical analysis

Apparent animal-level seroprevalence was calculated by dividing the number of LSDV positive animals by the total number of animals tested while herd-level prevalence was determined by dividing positive herds by total number of herds. A herd would be considered positive if at least one animal tested seropositive for lumpy skin disease. To calculate true seroprevalence, the apparent seroprevalence (AP) was adjusted for sensitivity (Se) and specificity (Sp) of ELISA test (91% and 99.7%, respectively). True prevalence was used to report seroprevalence at the animal-level for within-herd, regional, and national seroprevalences. True prevalence was calculated using the formula by Stevenson 2007 [50]:

$$ True\ prevalence=\frac{AP+ Sp-1}{Se+ Sp-1} $$

Although we do not anticipate a seasonal trend for serological data, we evaluated potential seasonality by plotting within-herd seroprevalence by month (Additional file 2). Possible risk factors for seropositivity were selected using univariable mixed effect logistic regression analysis with herd ID as a random effect to account for clustering at herd level, and all variables that were significant in the univariable analysis (*p* < 0.2) were further checked for co-linearity before multivariable analysis. During multivariable mixed effect logistic regression analysis, all non-significant variables were removed sequentially by backward elimination where the model with the lowest Akaike Information Criterion (AIC) value was chosen as the best model. At every step during model development, confounding was assessed by checking for changes in parameter estimates, and changes > 25% were considered to indicate confounding. To compare differences in animal level prevalence in the four regions of Uganda we performed a logistic regression with region as a categorical variable and herd as a random effect. In all the analyses performed, confidence levels were calculated at 95%, and a *P* value < 0.05 was used for statistical significance level, except for univariable analysis. Data analysis was done using STATA 2010, version 16 software.

## Additional files

Additional file 1: Map of Uganda showing districts sampled during the FMD sero-survey (blue) and sampling sites for LSD (yellow circle) (Source of map: This study). (DOCX 275 kb)

Additional file 2: Graph showing within herd true seroprevalence for all sampled herds and month when sampling was done. (DOCX 39 kb)

Additional file 3: Herd/village level seroprevalence of LSD in Uganda. (DOCX 19 kb)

## Data Availability

The datasets used and/or analysed during the current study are available from the corresponding author on reasonable request.

## Abbreviations

COVAB: College of Veterinary Medicine Animal Resources and Biosecurity
LSD: Lumpy Skin Disease
LSD: Lumpy Skin Disease Virus
MAAIF: Ministry of Agriculture Animal Industry and Fisheries
