# Supplementary figures and images for: Seroprevalence and risk factors for lumpy skin disease virus seropositivity in cattle in Uganda
Source: BMC Vet Res. 2019 Jul 8;15:236. doi: 10.1186/s12917-019-1983-9 (PMC6615106; doi:10.1186/s12917-019-1983-9)

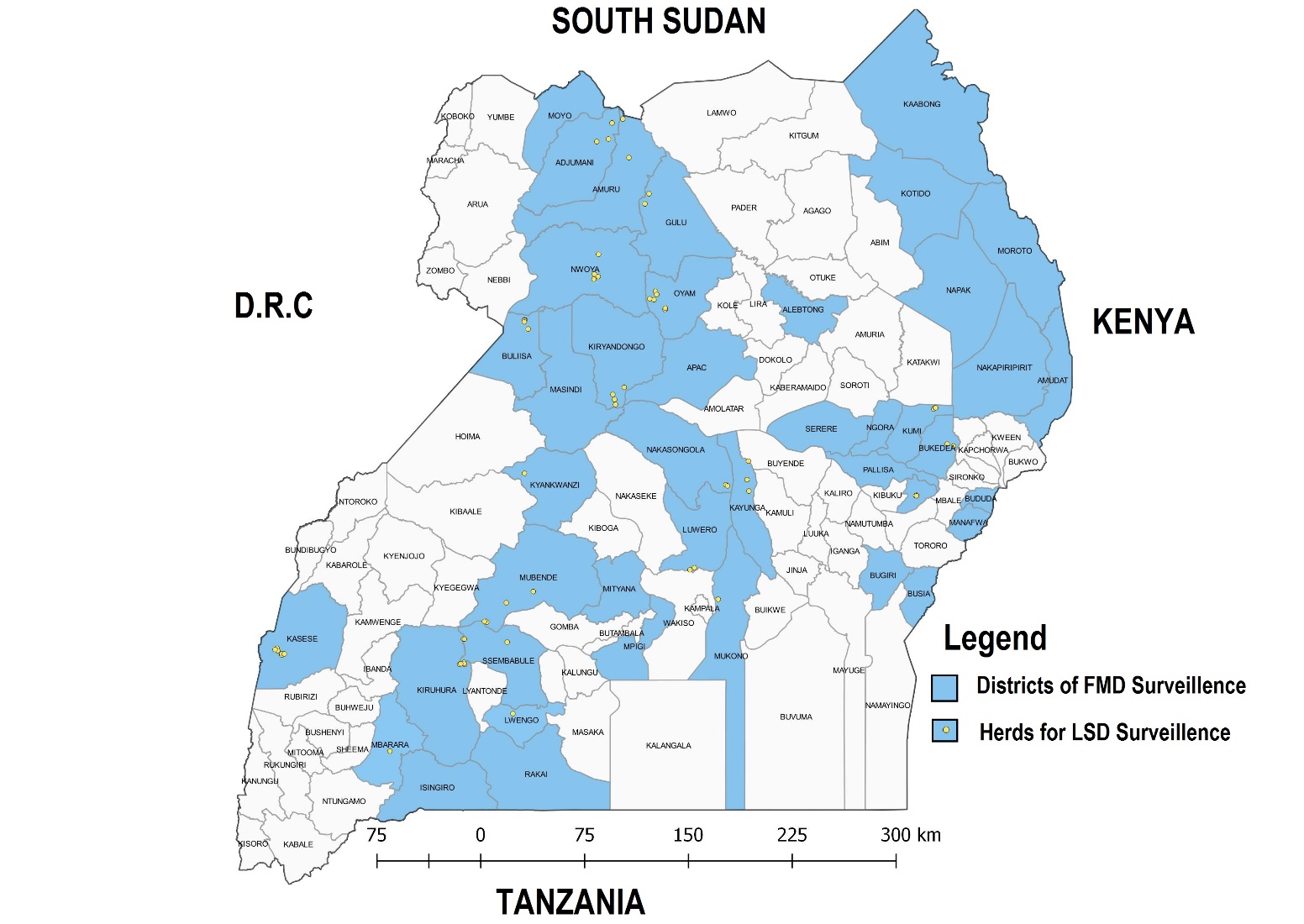

Supplement: Supplementary file 1 — Map of Uganda showing districts sampled during the FMD sero-survey (blue) and sampling sites for LSD (yellow circle) (Source of map: This study). (DOCX 275 kb) [file 12917_2019_1983_MOESM1_ESM.docx]
