# Supplementary material for: Seroprevalence and risk factors for lumpy skin disease virus seropositivity in cattle in Uganda
Source: BMC Vet Res. 2019 Jul 8;15:236. doi: 10.1186/s12917-019-1983-9 (PMC6615106; doi:10.1186/s12917-019-1983-9)
Supplement: Supplementary file 3 — Herd/village level seroprevalence of LSD in Uganda. (DOCX 19 kb) [file 12917_2019_1983_MOESM3_ESM.docx]

| Herd Name/Region | Number of cattle tested | Herd Size | Number Positive | Apparent Prevalence (%) | True Prevalence (%) | 95% CI |
| --- | --- | --- | --- | --- | --- | --- |
| Central Region |  |  |  |  |  |  |
| Kiyange | 43 | 300 | 14 | 32.6 | 35.6 | 19.1-48.5 |
| Bbaale | 43 | 250 | 12 | 27.9 | 30.4 | 15.3-43.7 |
| Kasokwe-kiryola | 43 | 400 | 9 | 20.9 | 22.7 | 10.0-36.0 |
| Lyakabirizi/Kiwogo | 20 | 400 | 2 | 10.0 | 10.7 | 1.2-31.7 |
| Kasambya-kyakasa | 43 | 240 | 2 | 4.7 | 4.9 | 0.6-15.8 |
| Kasambya-kyakasa II | 43 | 43 | 1 | 2.3 | 2.2 | 0.1-12.3 |
| Kasambya-kyakasa III | 43 | 108 | 3 | 7.0 | 7.4 | 1.5-19.1 |
| Kitenga I | 20 | 96 | 1 | 5.0 | 5.2 | 0.1-24.9 |
| Kitenga II | 43 | 80 | 2 | 4.7 | 4.9 | 0.6-15.8 |
| Nyimbwa | 30 | 41 | 1 | 3.3 | 3.3 | 0.1-17.2 |
| Namayiga west | 30 | 40 | 1 | 3.3 | 3.3 | 0.1-17.2 |
| Seeta town council | 40 | 45 | 11 | 27.5 | 30.0 | 14.6-43.9 |
| Namalinda I | 43 | 79 | 2 | 4.7 | 4.9 | 0.6-15.8 |
| Namalinda II | 43 | 61 | 2 | 4.7 | 4.9 | 0.6-15.8 |
| Eastern Region |  |  |  |  |  |  |
| Namusita-kakule | 23 | 65 | 2 | 8.7 | 9.3 | 1.1-28.0 |
| Namusita-kakule II | 59 | 99 | 2 | 3.4 | 3.4 | 0.4-11.7 |
| Namusita-kakule III | 86 | 81 | 8 | 9.3 | 9.9 | 4.1-17.5 |
| Aceere/kamutur | 100 | 136 | 5 | 5.0 | 5.2 | 1.6-11.3 |
| Ochus/kamutur | 95 | 130 | 11 | 11.6 | 12.5 | 5.9-19.8 |
| Tisai-tisai island | 77 | 164 | 11 | 14.3 | 15.4 | 7.4-24.1 |
| Tisai-tisai island II | 30 | 63 | 5 | 16.7 | 18.1 | 5.6-34.7 |
| Tisai-tisai island III | 72 | 80 | 8 | 11.1 | 11.9 | 4.9-20.7 |
| Northern Region |  |  |  |  |  |  |
| Pawinyo | 32 | 172 | 1 | 3.1 | 3.1 | 0.1-16.2 |
| Aringapi- ituji | 32 | 126 | 4 | 12.5 | 13.5 | 3.5-29.0 |
| Ituji- oloboo | 32 | 37 | 2 | 6.3 | 6.6 | 0.8-20.8 |
| Bibia-elegu | 32 | 100 | 4 | 12.5 | 13.5 | 3.5-29.0 |
| Elegu-atiak | 32 | 114 | 1 | 3.1 | 3.1 | 0.1-16.2 |
| Pawel adak | 32 | 269 | 0 | 0.0 | 0.0 | 0.0-0.0 |
| Pawel-adak | 32 | 46 | 2 | 6.3 | 6.6 | 0.8-20.8 |
| Pawel-parie | 32 | 50 | 0 | 0.0 | 0.0 | 0.0-0-0 |
| Agung | 32 | 78 | 0 | 0.0 | 0.0 | 0.0-0.0 |
| Todora-agung | 32 | 57 | 2 | 6.3 | 6.6 | 0.8-20.8 |
| Ceke- bidati | 32 | 214 | 0 | 0.0 | 0.0 | 0.0-0.0 |
| Todora-wipolo | 32 | 84 | 6 | 18.8 | 20.4 | 7.2-36.4 |
| Pukicha | 20 | 24 | 5 | 25.0 | 27.2 | 8.7-49.1 |
| Opat-oyer | 32 | 73 | 1 | 3.1 | 3.1 | 0.1-16.2 |
| Opat-oyer II | 32 | 64 | 3 | 9.4 | 10.0 | 2.0-25.0 |
| Acimi/aminonge | 20 | 28 | 0 | 0.0 | 0.0 | 0.0-0.0 |
| Argentina | 32 | 79 | 0 | 0.0 | 0.0 | 0.0-0.0 |
| Zuma-zambia | 32 | 62 | 1 | 3.1 | 3.1 | 0.1-16.2 |
| Zuma-ober | 24 | 89 | 0 | 0.0 | 0.0 | 0.0-0.0 |
| Western Region |  |  |  |  |  |  |
| Ngweno-nile | 24 | 96 | 1 | 4.2 | 4.3 | 0.1-21.1 |
| Ngweno-kisangye | 24 | 88 | 0 | 0.0 | 0.0 | 0.0-0.0 |
| Ngweno-kasinye | 24 | 62 | 2 | 8.3 | 8.8 | 1.0-27.0 |
| Ngwedo | 20 | 64 | 0 | 0.0 | 0.0 | 0.0-0.0 |
| Kirama | 24 | 170 | 0 | 0.0 | 0.0 | 0.0-0.0 |
| Kirama II | 92 | 456 | 13 | 14.1 | 15.2 | 7.7-23.0 |
| Nyakatonzi | 30 | 380 | 6 | 20.0 | 21.7 | 7.7-38.6 |
| Nyakatonzi II | 24 | 141 | 0 | 0.0 | 0.0 | 0.0-0.0 |
| Kamuruli | 20 | 120 | 3 | 15.0 | 16.2 | 3.2-37.9 |
| Kagongo II | 20 | 93 | 0 | 0.0 | 0.0 | 0.0-0.0 |
| Kagongo III | 24 | 109 | 0 | 0.0 | 0.0 | 0.0-0.0 |
| Kagongo I | 24 | 74 | 0 | 0.0 | 0.0 | 0.0-0.0 |
| Kasana | 20 | 65 | 0 | 0.0 | 0.0 | 0.0-0.0 |
| Orubaale | 24 | 50 | 1 | 4.2 | 4.3 | 0.1-21.1 |
| Kinoni/rubaale | 24 | 35 | 2 | 8.3 | 8.8 | 1.0-27.0 |
| Kasaana | 20 | 56 | 2 | 10.0 | 10.7 | 1.2-31.7 |
| Kishebashebe | 20 | 37 | 0 | 0.0 | 0.0 | 0.0-0.0 |
| Burunga/buyaga | 20 | 36 | 0 | 0.0 | 0.0 | 0.0-0.0 |
| Kiguma/bwantama | 24 | 24 | 1 | 4.2 | 4.3 | 0.1-21.1 |
| Wakisanyi | 20 | 59 | 0 | 0.0 | 0.0 | 0.0-0.0 |
| Wakisanyi II | 24 | 61 | 1 | 4.2 | 4.3 | 0.1-21.1 |
| Wakisanyi III | 24 | 44 | 1 | 4.2 | 4.3 | 0.1-21.1 |
| Kitwara | 24 | 110 | 1 | 4.2 | 4.3 | 0.1-21.1 |
| Rukindo/nyakayojo | 24 | 52 | 4 | 16.7 | 18.1 | 4.7-37.4 |
| Total | **2263** |  | **185** | **8.2** | 8.7 | 7.1-9.4 |
