# Supplementary material for: Seroprevalence and risk factors for lumpy skin disease virus seropositivity in cattle in Uganda
Source: BMC Vet Res. 2019 Jul 8;15:236. doi: 10.1186/s12917-019-1983-9 (PMC6615106; doi:10.1186/s12917-019-1983-9)
Supplement: Supplementary file 3 [file 12917_2019_1983_MOESM2_ESM.docx]

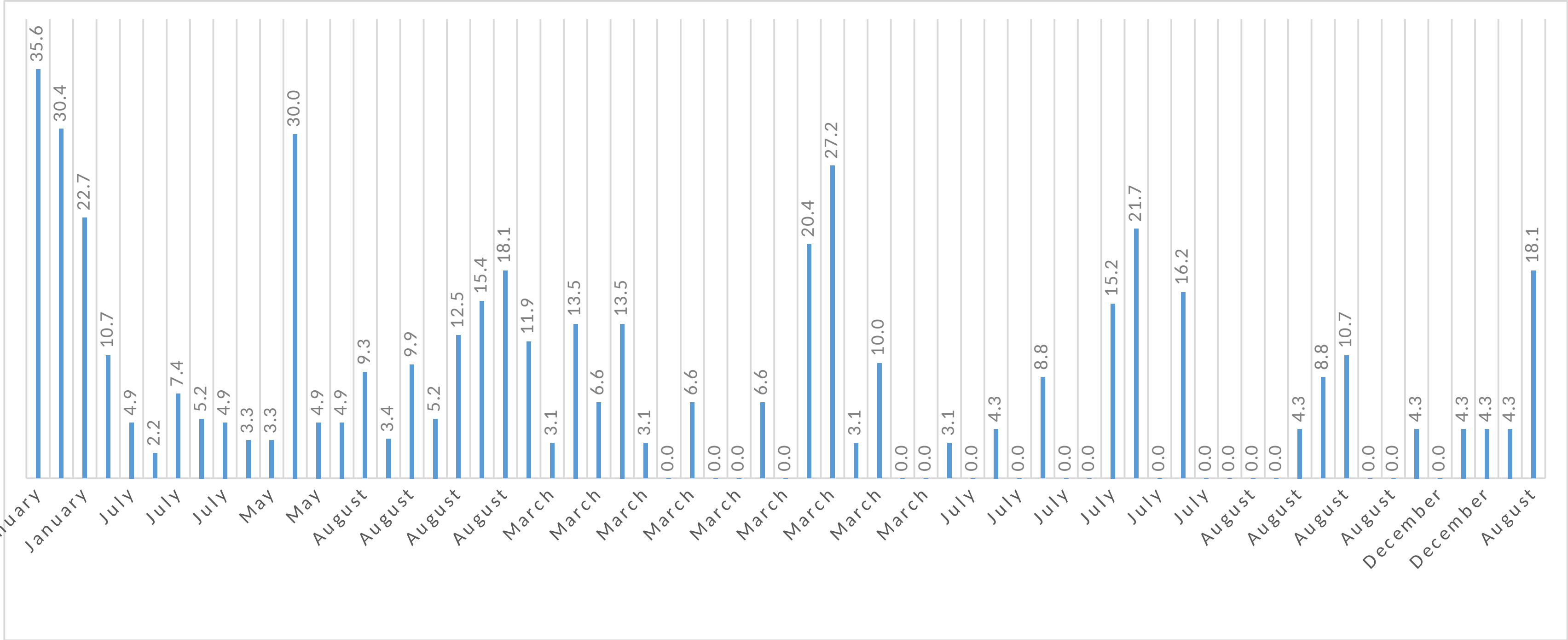
35.6
30.4
22.7
10.7
4.9
2.2
7.4
5.2
4.9
3.3
3.3
30.0
4.9
4.9
9.3
3.4
9.9
5.2
12.5
15.4
18.1
11.9
3.1
13.5
6.6
13.5
3.1
0.0
6.6
0.0
0.0
6.6
0.0
20.4
27.2
3.1
10.0
0.0
0.0
3.1
0.0
4.3
0.0
8.8
0.0
0.0
15.2
21.7
0.0
16.2
0.0
0.0
0.0
0.0
4.3
8.8
10.7
0.0
0.0
4.3
0.0
4.3
4.3
4.3
18.1
January
January
July
July
July
May
May
August
August
August
August
March
March
March
March
March
March
March
March
March
July
July
July
July
July
July
August
August
August
August
December
December
August
